# Chronic activation of 4-1BB signaling induces granuloma development in tumor-draining lymph nodes that is detrimental to subsequent CD8^+^ T cell responses

**DOI:** 10.1038/s41423-020-00533-3

**Published:** 2020-08-31

**Authors:** Seon-Hee Kim, Rohit Singh, Chungyong Han, Eunjung Cho, Yu I. Kim, Don G. Lee, Young H. Kim, Sang Soo Kim, Dong Hoon Shin, Hye Jin You, Hyeon-Woo Lee, Byoung S. Kwon, Beom K. Choi

**Affiliations:** 1 Division of Tumor Immunology, National Cancer Center, Goyang, 10408 Republic of Korea; 2 Graduate School of Cancer Science and Policy, National Cancer Center, Goyang, 10408 Republic of Korea; 3 Biomedicine Production Branch, Program for Immunotherapy Research, Goyang, 10408 Republic of Korea; 4 Eutilex Institute for Biomedical Research, Eutilex, Co., Ltd., Seoul, 08594 Republic of Korea; 5 Division of Convergence Technology, National Cancer Center, Goyang, 10408 Republic of Korea; 6 Division of Translational Science, National Cancer Center, Goyang, 10408 Republic of Korea; 7 Institute of Oral Biology, School of Dentistry, Graduate School, Kyung Hee University, Seoul, 02447 Republic of Korea; 8 Department of Medicine, Tulane University Health Sciences Center, New Orleans, LA 70112 USA

**Keywords:** 4-1BB, Costimulation, Granuloma, CD8 lymphocyte, Macrophage, Immunosuppression, Lymphocyte activation

## Abstract

The antitumor capabilities of agonistic anti-4-1BB mAbs have made them an attractive target for tumor immunotherapy. However, the adverse side effects associated with agonist antibodies have hindered their clinical development. Here, we aimed to study the immune-related adverse events of repeated doses and long-term use of agonistic anti-4-1BB mAbs. We show that chronic activation of 4-1BB signals induced the accumulation of IFN-γ-producing PD-1^+^CD8^+^ T cells in the secondary lymphoid organs of tumor-bearing mice by increasing the number of dividing CD8^+^ T cells, which was beneficial for suppressing tumor growth in the early phase of anti-4-1BB induction. However, repeated exposure to anti-4-1BB mAbs led to granuloma development in tumor-draining lymph nodes (TDLNs) of mice due to recruitment and accumulation of macrophages via the CD8^+^ T cell-IFN-γ axis. This was accompanied by excessive lymph node swelling, which impaired the sequential activation of CD8^+^ T cells. Our data provide insights into the immune-related adverse events of long-term agonist 4-1BB antibody dosing, which should be considered during the clinical development of immunomodulating therapy.

## Introduction

Immune checkpoint blockades (ICBs) targeting CTLA-4 and PD-1/PD-L1 have been approved for the treatment of diverse cancers.^1^ Mechanistically, PD-1-targeting ICBs have been considered to enhance T cell responses, particularly in tumor tissues, as a higher number of PD-1^+^ tumor-infiltrating lymphocytes (TILs) and/or PD-L1^high^ tumor tissues are associated with better clinical responses.^2,3^ However, certain models suggest that PD-1 blockades primarily act in tumor-draining lymph nodes (TDLNs) since PD-1-mediated T cell inhibition requires CD28, which is abundantly expressed on T cells in lymphoid tissues^4^; however, cancer patients lack CD28 expression on CD8^+^ T cells in the tumor microenvironment.^5,6^ PD-1 blockade not only enhances antitumor immunity but also induces a broad spectrum of autoimmune-like reactions, including pneumonitis, hepatitis, and dermatitis.^7^ Moreover, several clinical reports indicate that some cancer patients with good oncologic responses to PD-1 blockade develop lymphatic sarcoidosis, an inflammatory disease characterized by granulomas in the lung, skin, and lymph nodes (LNs).^8–10^ However, LNs are the most frequently involved site. Moreover, cancer patients who have developed sarcoidosis following ICB treatment tend to have hyperimmune responses,^11^ while patients who develop sarcoidosis in an immunotherapy-independent manner appear to exhibit hypoimmune responses.^12^ The development of such granulomatous/sarcoid-like lesions following administration of ICBs, including CTLA-4 and PD-1/PD-L1 blockades, is significant because they mimic disease recurrence and occasionally lead to discontinuation of therapy.^10^ Therefore, the recognition of this type of immune-related adverse effect (irAE) becomes important for effective treatment of cancer patients.

The immune checkpoint modulator 4-1BB (CD137; TNFRSF9) is constitutively expressed on Foxp3^+^ regulatory T cells and dendritic cells (DCs) at low levels^13–15^ and temporarily expressed on activated NK, NKT, DCs, and T cells.^16^ Previous studies have characterized 4-1BB signals, particularly on T cells; the 4-1BB signal preferentially enhances the responses of CD8^+^ T cells in vitro and in vivo,^17,18^ prevents activation-induced cell death (AICD),^19^ promotes cell cycle progression and Th1 responses,^20,21^ and accelerates metabolism.^22,23^ As triggering of the 4-1BB signal on CD8^+^ T cells has therapeutic potential in cancer,^16^ agonistic anti-4-1BB mAbs are under active investigation as immunotherapeutic agents.^24,25^ Of note, agonistic anti-4-1BB mAbs primarily boost T cell responses in TDLNs and are associated with a series of lymphatic anomalies, including lymphadenopathy and splenomegaly.^26,27^ Considering that TDLN is the primary site of 4-1BB activation, such anomalies may affect the therapeutic efficacy of immunomodulatory agents.

In this study, we sought to characterize the effects of repeated doses and long-term use of agonistic anti-4-1BB mAbs in cancer immunotherapy to reflect the observations in a clinical setting.^24,25^ We provide evidence that chronic 4-1BB triggering leads to excessive LN swelling along with granuloma formation in TDLNs and describe the cellular mechanism associated with granuloma formation and its impacts on anti-4-1BB therapy.

## Materials and methods

### Reagents and antibodies

Human gp100_25–33_ (hgp100, KVPRNQDWL) peptides were synthesized by Peptron (Daejeon, Korea). CD8 microbeads were purchased from Miltenyi Biotec (Auburn, CA). All antibodies used for flow cytometry were purchased from BD Bioscience. Rat IgG and anti-CD8 mAb (2.43) were purchased from BioXCell (West Lebanon, NH). Agonistic anti-4-1BB mAb was purified from a hybridoma (3E1, originally obtained from Dr Robert Mittler, Emory University) using protein G-agarose. Anti-mouse IFN-γ-PE, CD8β-PE-Cy5, CD4-FITC, PD-1-PE, PD-1-PE-Cy5, CD8β-PE, CD8β-APC, CD62L-PE, KLRG-1-PE, LAG3-PE, TIM-3-PE, Thy1.1-FITC, Thy1.1-PE-Cy5, CD45-APC, and 7-AAD for flow cytometry were purchased from BD Bioscience. Anti-PCNA mAb was obtained from Dako, ER-TR7-Dylight488 was obtained from Novus, anti-CD68-biotin was obtained from Miltenyi Biotec, anti-rat IgG-FITC and anti-rat IgG-Cy3 were obtained from Jackson Lab, anti-mouse CD8β (Ly-3)-Alexa 647, anti-B220 (RA3-6B2)-Alexa 594, anti-PD-1-FITC, and streptavidin-Cy3 were obtained from Biolegend, and CellTrace Violet and CellTrace carboxyfluorescein succinimidyl ester (CFSE) were obtained from ThermoFisher Scientific.

### Mice

Six-to-eight-week-old C57BL/6 female mice were purchased from OrientBio (Gapyeong, Korea), and B6.Cg-Thy1^a^/Cy Tg(TcraTcrb)8Rest/J (pmel-1 Thy1.1^+^) transgenic (Tg), B6.129S7-Ifngr1^tm1Agt^/J (IFN-γR^−/−^) and B6.129P2-*S1pr1*^tm1Hrose^/J (S1PR1-GFP) knock-in (KI) female mice were purchased from the Jackson Laboratory (JAX; Bar Harbor, ME). S1PR1-GFP × Thy1.1^+^pmel-1 Tg mice were generated by crossing S1PR1-GFP KI mice with pmel-1 Thy1.1^+^ Tg mice. All mice were maintained under specific-pathogen-free conditions in the animal facility of the National Cancer Center in Korea. Procedures (NCC-19-481) were approved by the Institutional Animal Care and Use Committee of the National Cancer Center Institute. Animal experiments were conducted according to the Guidelines on the Care and Use of Laboratory Animals from the Institute of Laboratory Animal Resources.

### MC38 tumor model, treatments, and flow cytometry

MC38 adenocarcinoma cells (5 × 10^5^ cells per mouse) were injected subcutaneously (s.c.) into the backs of C57BL/6 mice, and 100 μg of rat IgG and agonistic anti-4-1BB mAb were injected intraperitoneally (i.p.) into the mice every 5 days starting 7 days after tumor injection. For flow cytometry, single-cell suspensions were prepared from inguinal TDLNs and the spleen 12 days after tumor injection, and 1 × 10^6^ cells were incubated with Fc blocker (2.4G2 antibody) for 5 min and further stained with anti-CD4-FITC, anti-PD-1-PE, and anti-CD8β-PE-Cy5 for 30 min at 4 °C. For TIL analysis, tumor tissues were dissected and cut into 3–5 mm pieces on day 14, suspended in 5 ml RPMI 1640 medium supplemented with 0.1% collagenase IV and 100 U/ml DNase I, and placed on a shaking incubator for 30 min at 37 °C. Single-cell suspensions were stained with anti-CD4-FITC, anti-PD-1-PE, and anti-CD8β-PE-Cy5 for 30 min at 4 °C. All samples were subsequently analyzed with a FACSCalibur (BD Bioscience).

### Confocal microscopy

For confocal microscopy of TDLNs, inguinal LNs were collected from each group of mice, embedded in OCT compound, and used to prepare frozen sections (10 μm thickness). Frozen sections of rat IgG- and anti-4-1BB-treated TDLNs were stained with the anti-PD-1 mAbs (29F.1A12)-FITC to detect PD-1^+^ cells, anti-ER-TR7-DyLight to visualize the LN structure, and anti-mouse CD8β (Ly-3)-Alexa 647 and anti-B220 (RA3-6B2)-Alexa 594 to visualize the T and B cell zones.

Alternatively, inguinal TDLNs were fixed with 4% paraformaldehyde and embedded in OCT compound. Twenty-micrometer frozen sections were cut, blocked with a Streptavidin/Biotin Blocking kit (Vector, SP-2002), stained with anti-mouse CD68-biotin (FA-11) and detected with streptavidin-Cy3 or streptavidin-Cy5. Slides were mounted with DAPI-containing solution. Z-stack images were acquired with a laser scanning microscope (Zeiss LSM780, Carl Zeiss, Germany) and projected as a maximum intensity projection.

### PCNA staining of TDLN

MC38 tumor cells were subcutaneously injected into C57BL/6 mice, and rat IgG or anti-4-1BB mAb was administered four times every 5 days starting on day 10 of the tumor cell injection. TDLNs were collected from the mice 3 days after the 4th injection of Ab and fixed with 10% formalin solution. Fixed TDLNs were embedded in paraffin wax, and 5 mm sections were cut. The slides were stained with anti-PCNA mAb (Dako), followed by staining with HRP-conjugated secondary Ab and colorization with DAB chromogen.

### Trafficking of proliferating pmel-1 Thy1.1^+^CD8^+^ T cells in B16-F10 melanoma-bearing mice

C57BL/6 mice were injected subcutaneously with 2 × 10^5^ B16-F10 melanoma cells. Seven days after tumor injection, CD8^+^ T cells were isolated from pmel-1 Thy1.1^+^ Tg mice using CD8 microbeads (Miltenyi Biotec), labeled with 10 μM CFSE using the CellTrace CFSE Cell Proliferation kit (Thermo Scientific^TM^), and injected intravenously into the mice (2 × 10^6^ cells per mouse). The mice were subsequently immunized subcutaneously with 20 μg of hgp100 peptide emulsified with incomplete Freund’s adjuvant (IFA, Thermo Scientific^TM^) and intraperitoneally injected with 100 μg of anti-4-1BB mAb or rat IgG. For flow cytometry, single-cell suspensions were prepared from inguinal TDLNs and tumor tissues 4 or 7 days after Ab injection and stained with anti-Thy1.1-PE and anti-CD8-PE-Cy5 for 30 min at 4 °C. All samples were subsequently analyzed with a FACSCalibur (BD Bioscience).

To examine the effect of anti-4-1BB triggering on T cell egress, C57BL/6 mice were injected subcutaneously with 2 × 10^5^ B16-F10 melanoma cells. On day 7, the mice were injected intravenously with CFSE-labeled pmel-1 Thy1.1^+^CD8^+^ T cells (5 × 10^6^ cells per mouse) and immunized subcutaneously with the hgp100 peptide in IFA as described above. Anti-4-1BB mAb or rat IgG was administered to the mice on days 7 and 9. Flow cytometry was performed on days 11 and 14 by staining single-cell suspensions of inguinal TDLNs and tumor tissues with anti-Thy1.1-PE and anti-CD8-PE-Cy5 for 30 min at 4 °C. All samples were subsequently analyzed with a FACSCalibur (BD Bioscience).

### Adoptive transfer of naive pmel-1 CD8^+^ T cells to mAb-pretreated and tumor-bearing mice

C57BL/6 mice were injected subcutaneously with 2 × 10^5^ B16-F10 melanoma cells and further treated intraperitoneally with 100 μg of anti-4-1BB mAb or rat IgG on days 5 and 10. CFSE-labeled naive pmel-1 Thy1.1^+^CD8^+^ T cells were prepared as described above and injected intravenously into the mAb-pretreated mice on day 14. Inguinal TDLNs were collected from the mice 1 or 7 days after pmel-1 CD8^+^ T cell transfer and stained with anti-Thy1.1-PE and anti-CD8-PE-Cy5. Alternatively, single-cell suspensions of tumor tissues were prepared on day 7, stained with anti-Thy1.1-PE and anti-CD8-APC antibodies, and further stained with 7-AAD. All samples were subsequently analyzed with a FACSCalibur (BD Bioscience).

### Growth rate of MC38 tumor cells in mAb-pretreated and OVA-immunized mice

C57BL/6 mice were immunized subcutaneously with 20 μg of OVA emulsified in IFA and injected with 100 μg of rat IgG or anti-4-1BB mAb on days 3, 6, and 9. On day 20, 5 × 10^5^ MC38 tumor cells were injected subcutaneously into the untreated (UnTx) or mAb+OVA-treated (Ab-Tx) mice, which were further injected with rat IgG or anti-4-1BB mAb every 5 days starting 3 days after the tumor challenge. Tumor growth rates were monitored every 3–4 days.

### Calculation of absolute cell numbers

Total viable cell numbers were assessed by counting the single-cell suspensions of TDLNs with an ADAM-MC2 cell counter (NanoEnTek, Seoul, Korea) according to the manufacturer’s instructions, and the absolute numbers of each population were calculated by multiplying the percentage of measured cells by the total number of viable cells (absolute number = % of measured cells × total cells recovered).

### Statistical analysis

All data were analyzed with the statistical program Prism GraphPad 7.0 (San Diego, CA, USA). Student’s *t* test was used to determine the statistical significance of differences.

## Results

### Long-term treatment with anti-4-1BB mAb induces granuloma formation in TDLNs

**Fig. 1:**
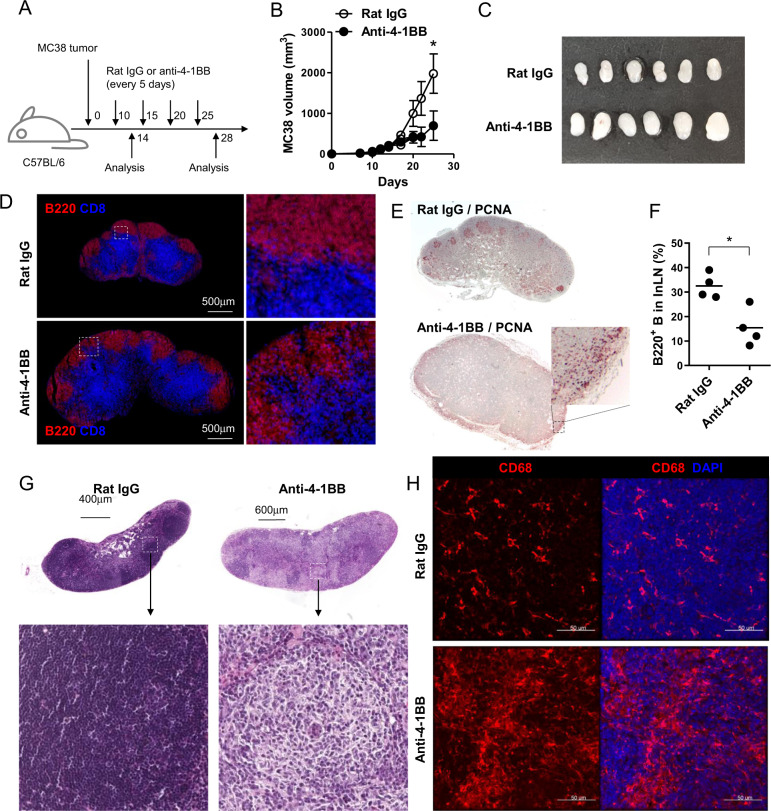
Granulomas of TDLNs following the repeated injection of anti-4-1BB mAb in vivo. **a** C57BL/6 mice were injected subcutaneously with MC38 tumor cells and received anti-4-1BB mAb or rat IgG every 5 days, for a total of four times, from day 10. **b** Growth rate of MC38 tumors. **c** Inguinal TDLNs on day 14. **d** Frozen sections were prepared from TDLNs on day 14 and stained with anti-mouse CD8β-Alexa 647 and anti-B220-Alexa 594 antibodies. Slides were mounted with DAPI-containing solution. **e** Three days after 4th mAb injection, TDLNs were collected, and the paraffin sections of TDLNs were stained with anti-PCNA mAb and HRP-conjugated 2nd Ab. **f** Percentages of B220^+^ B cells in TDLNs on day 28. **g** H&E staining of paraffin sections from rat IgG- or anti-4-1BB-treated TDLNs on day 28. **h** Frozen sections of inguinal TDLNs on day 28 were stained with anti-mouse CD68-biotin and detected using streptavidin-Cy3. Slides were mounted with DAPI-containing solution. Confocal images were captured by a laser scanning microscope (Zeiss LSM780, Carl Zeiss). Data are from two (**c**, **d**) and three (**b**, **e**–**h**) independent experiments with 3–5 mice per experiment. Student’s *t* test was performed in **b** and **f**, and the results are shown as the means ± SDs (*p < 0.05; **p < 0.01)

4-1BB triggering preferentially enhances the proliferation of CD8^+^ T cells, and thus, agonistic anti-4-1BB mAb was repeatedly administered to tumor-bearing mice to enhance antitumor CD8^+^ T cell levels.^17,28^ However, since enhanced CD8^+^ T cell responses due to 4-1BB triggering suppress B and CD4^+^ T cells in the LNs and cause lymphadenopathy,^26,27^ to study the effect of the repeated injection of anti-4-1BB mAb on LN structure, we injected MC38 tumor cells subcutaneously into the back of C57BL/6 mice, and subsequently, rat IgG or anti-4-1BB mAb was administered intraperitoneally to the mice every 5 days starting on day 10 after tumor injection (Fig. 1a). It was observed that 4-1BB triggering significantly suppressed tumor growth (Fig. 1b). When the inguinal TDLNs were collected from the mice 4 days after a single injection of rat IgG or anti-4-1BB mAb, enhanced LN swelling was observed in anti-4-1BB-triggered mice compared to that in the rat IgG-treated mice (Fig. 1c). We next prepared frozen sections of these TDLNs and stained them with anti-CD8β and anti-B220 mAb to visualize the T and B cell zones. The confocal images showed that CD8^+^ T and B cells were clearly separated in rat IgG-treated LNs, while the B cell zones were reduced in size in the anti-4-1BB-treated LNs due to the expansion of the CD8^+^ T cell area, and the boundary of the T and B cell zones was blurred (Fig. 1d).

When the mice were repeatedly exposed to anti-4-1BB mAb every 5 days at least four times, the germinal centers, including PCNA^+^ B cells, completely disappeared in anti-4-1BB-treated TDLNs, and most of the PCNA^+^ cells were located in a marginal region of anti-4-1BB-treated TDLNs; however, the germinal centers were intact and normally developed in rat IgG-treated TDLNs (Fig. 1e). Consequently, the B cells were significantly decreased in anti-4-1BB-treated TDLNs compared with those in rat IgG-treated mice (Fig. 1f), as previously reported.^25^ H&E staining showed that the TDLNs of rat IgG-treated mice showed typical dark areas due to the enrichment of lymphocytes with dense chromatin, while multiple bright areas enriched with myeloid cells were found in anti-4-1BB-treated TDLNs, indicating the occurrence of the granulomatous reaction (Fig. 1g). Since macrophages are the major population found in granulomas,^29^ we stained the TDLN sections with anti-CD68 mAb and found that a large proportion of the CD68^+^ macrophages in anti-4-1BB-treated TDLNs were in clustered form compared with those in rat IgG-treated mice (Fig. 1h). Anti-F4/80 staining also showed abundant F4/80^+^ macrophages in anti-4-1BB-treated TDLNs compared with those in rat IgG-treated mice (Supplementary Fig. 1).

An increase in CD68^+^ macrophages within inguinal TDLNs was also observed in B16-F10 mouse melanoma-bearing mice following repeated injection of anti-4-1BB mAb (Supplementary Fig. 2). Moreover, when the inguinal TDLNs as well as cervical or axillary LNs, which served as non-TDLNs, from MC38-bearing mice were examined for LN swelling, CD68^+^ macrophages were increased, and B cell zone shrinkage was not evident in non-TDLNs compared to that in TDLNs (Supplementary Fig. 3). These findings indicate that these properties of 4-1BB triggering in vivo were dependent on an increase in activated T cells in TDLNs. Collectively, our data suggest that long-term treatment with anti-4-1BB mAb in vivo induced excessive expansion of activated CD8^+^ T cells in TDLNs, led to a decrease in B cells by shrinking B cell zones as previously reported,^27^ and induced the development of granulomas by enhancing the accumulation of macrophages in the TDLN.

### 4-1BB triggering induces granuloma formation in TDLNs via the CD8^+^ T cell-IFN-γ axis

**Fig. 2:**
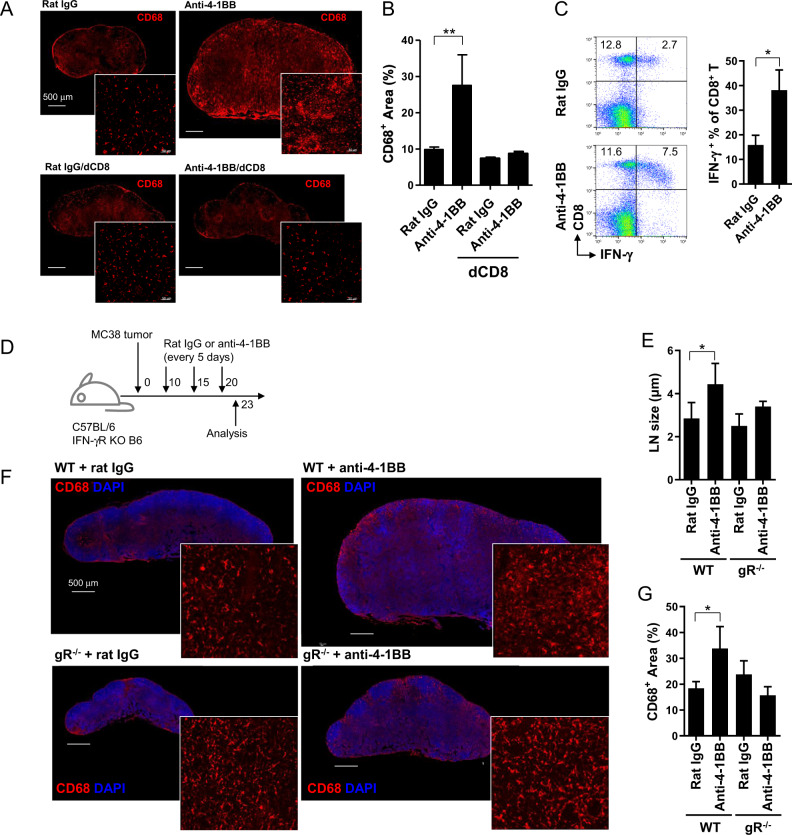
4-1BB-induced LN granulomas in the absence of CD8^+^ T cells or IFN-γ. **a** MC38 tumor-bearing mice were injected intraperitoneally with rat IgG or anti-4-1BB mAb, and half of the mice were further treated with depleting anti-CD8 mAb (dCD8) 10 and 15 days after injection of MC38 tumor cells. On day 23, frozen sections of TDLNs were stained with anti-mouse CD68-biotin and detected using streptavidin-Cy3. **b** CD68^+^ areas were calculated from (**a**) using ImageJ. **c** IFN-γ expression of rat IgG- or anti-4-1BB-treated CD8^+^ T cells on day 18. **d**–**g** WT and IFN-γR KO (gR^−/−^) B6 mice were injected subcutaneously with MC38 tumor cells and treated with rat IgG or anti-4-1BB mAb every 5 days from day 10 as described above (**d**). **e** On day 23, LN longitudinal length was calculated using a stereomicroscope (Zeiss Stereo Discovery V20) with a camera (Zeiss AxioCamHRc camera). **f** Frozen sections of TDLNs on day 23 were stained with anti-mouse CD68-biotin and detected using streptavidin-Cy3. Slides were mounted with DAPI-containing solution. Confocal images were captured by a laser scanning microscope (Zeiss LSM780, Carl Zeiss). **g** CD68^+^ areas were calculated from (**f**) using ImageJ. Data are from two (**c**–**g**) and three (**a**, **b**) independent experiments with 3–5 mice per experiment. Student’s *t* test was performed in **b**, **c**, **e**, and **g** and is shown as the means ± SDs (*p < 0.05; **p < 0.01)

Although granuloma formation is known to be mediated by IFN-γ secreted from CD4^+^ T cells,^29^ anti-4-1BB-induced granuloma development seems to be mediated by CD8^+^ T cells rather than CD4^+^ T cells, since 4-1BB triggering preferentially enhances the proliferation of and IFN-γ production by CD8^+^ T cells.^28^ To investigate whether granuloma formation is mediated by IFN-γ from CD8^+^ T cells, we assessed anti-4-1BB-mediated granuloma formation in mice by depleting anti-CD8 mAb 10–15 days after injection of MC38 tumor cells. The TDLNs were collected from the IgG- or anti-4-1BB mAb-treated MC38 tumor-bearing B6 mice after the 4th injection of Abs, and the TDLN sections were stained with anti-CD68 mAb. We observed an abundance of CD68^+^ macrophages and LN swelling in anti-4-1BB-treated TDLNs, which was completely reversed in anti-CD8-depleted mice (Fig. 2a, b).

The intracellular staining of the TDLN cells collected from MC38 tumor-bearing mice 4 days after a single injection of anti-IFN-γ mAb showed that the 4-1BB triggering increased the number of IFN-γ-producing CD8^+^ T cells by more than threefold compared with that of rat IgG-treated CD8^+^ T cells (Fig. 2c). To confirm these findings, we examined whether 4-1BB-induced granuloma formation was reversed in the absence of IFN-γ. To this end, MC38 tumor cells were injected into WT and IFN-γR (gR^−/−^)-deficient B6 mice and further treated with rat IgG or anti-4-1BB (Fig. 2d). When the TDLN sections collected at day 23 were stained with anti-CD68 mAb, CD68^+^ macrophages were abundantly found in anti-4-1BB-treated TDLNs in clustered form, which was ameliorated in anti-4-1BB-treated gR^−/−^ mice (Fig. 2e, f), even if IFN-γ production by gR^−/−^ CD8^+^ T cells was still significantly enhanced by 4-1BB triggering (Supplementary Fig. 4). Moreover, DAPI staining indicated that the B cell zones remained intact in anti-4-1BB-treated gR^−/−^ mice (Fig. 2e). Collectively, these results indicate that the repeated injection of anti-4-1BB mAb induced granuloma formation in TDLNs via the CD8^+^ T cell-IFN-γ axis.

### 4-1BB-triggered LN swelling accompanies the accumulation of PD-1^High^CD8^+^ T cells in the medulla region of TDLNs and alteration of the lymphatic structure

**Fig. 3:**
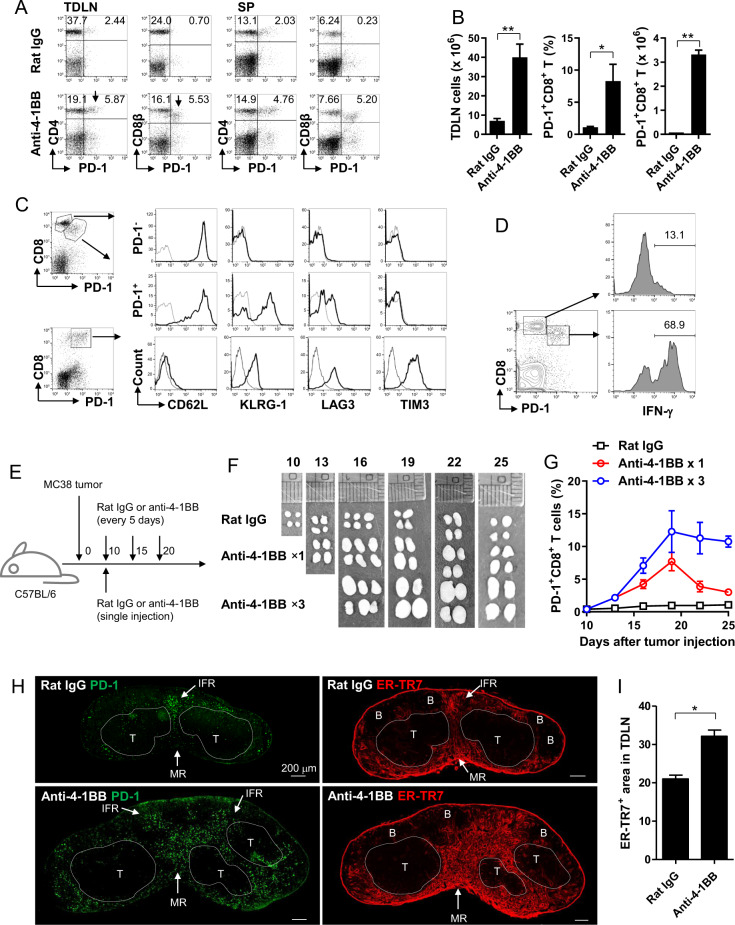
4-1BB triggering in vivo increases PD-1^+^CD8^+^ T cells in the medulla of TDLNs. **a**–**d** Rat IgG or anti-4-1BB mAb was injected i.p. to the mice 5 and 10 days after the challenge of MC38 tumor cells. Inguinal TDLN cells were collected from the mice on day 13. PD-1 expression on CD4^+^ T and CD8^+^ T cells in inguinal TDLNs and the spleen was analyzed by flow cytometry (**a**). Total live cell numbers and the percentage and absolute number of PD-1^+^CD8^+^ T cells in inguinal TDLNs were calculated (**b**). The phenotype of PD-1^+^CD8^+^ T cells in inguinal TDLNs and tumor tissues (**c**). IFN-γ expression of PD-1^−^ and PD-1^+^ CD8^+^ T cells in TDLNs of anti-4-1BB-treated mice (**d**). **e**–**i** C57BL/6 mice were single- or multiple-injected intraperitoneally with rat IgG or anti-4-1BB mAb from 10 days after MC38 tumor challenge (**e**). **f** Inguinal TDLNs collected and photographed on the indicated days. **g** TDLN cells were stained with anti-PD-1-PE and anti-CD8β-PE-Cy5 at the indicated days, and percentages of PD-1^+^CD8^+^ T cells were calculated. **h** Inguinal TDLNs were collected from each group of mice 5 days after a single injection of Ab. Frozen sections of TDLNs were stained with anti-PD-1 or anti-ER-TR7 mAb, followed by fluorescence-conjugated secondary mAb. Images were captured with a laser scanning microscope (Zeiss LSM780, Carl Zeiss). T T cell zone, B B cell zone, IFR interfollicular region, MR medullar region. **i** ER-TR7^+^ areas in TDLNs were calculated from (**h**) with the ImageJ program. Data are from three (**a**–**c**, **h**, **i**) or two (**d**, **f**, **g**) independent experiments with five mice per experiment. Student’s *t* test was performed in **b**, **g**, and **i** and is shown as the means ± SDs (*p < 0.05; **p < 0.01)

Our results showed that the CD8^+^ T cell-IFN-γ axis was essential for granuloma formation in TDLNs (Fig. 2). We next questioned the location of the activated IFN-γ-producing CD8^+^ T cells to determine where granuloma development was initiated in the TDLNs of 4-1BB-treated mice. It has been shown that activated CD8^+^ T cells increase the expression of PD-1,^30^ and thus, we first examined whether the ratio of activated T cells could be assessed by detecting PD-1 on T cells from TDLNs and the spleen. The results show that PD-1-expressing CD4^+^ and CD8^+^ T cells were only minimally detected in TDLNs and spleens in rat IgG-treated mice (Fig. 3a). However, 4-1BB triggering increased not only the ratio and number of PD-1-expressing T cells but also generated a unique population of PD-1^High^CD8^+^ T cells in secondary lymphoid organs (Fig. 3a, b). Since PD-1^High^CD8^+^ T cells were readily found in tumor tissues, we next compared the expression of several activation markers on PD-1^High^CD8^+^ T cells in 4-1BB-triggered TDLNs and tumor tissues. PD-1^High^CD8^+^ T cells in TDLNs were of the KLRG-1^+^LAG3^+^TIM3^−^ phenotype, with downregulated CD62L expression, while the PD-1^−^CD8^+^ T cells in TDLNs were of the CD62L^High^KLRG-1^−^LAG3^−^TIM3^−^ phenotype (Fig. 3c). The results indicate that the PD-1^High^CD8^+^ T cells in TDLNs were activated CD8^+^ T cells under proliferation mode. Moreover, when the 4-1BB-triggered TDLNs were intracellularly stained with anti-IFN-γ mAb, the PD-1^High^CD8^+^ T cells served as the major source of IFN-γ (Fig. 3d).

To examine the correlation between LN swelling and PD-1^High^CD8^+^ T cells, MC38 tumor-bearing mice were injected with rat IgG or anti-4-1BB mAb either once or multiple times, and the LN swelling and ratio of PD-1^+^CD8^+^ T cells were routinely assessed (Fig. 3e). LN swelling was temporary in the mice that received a single injection of anti-4-1BB mAb, as the LN size was decreased ~12 days after Ab injection; however, it was sustained in mice that received multiple injections of anti-4-1BB mAb (Fig. 3f). Consistent with these data, the ratio of PD-1^High^CD8^+^ T cells in TDLNs was also temporarily increased by a single injection of anti-4-1BB mAb, which was sustained for a longer period of time in the mice that received multiple injections of anti-4-1BB mAb (Fig. 3g).

Finally, to examine the localization of the activated PD-1^High^CD8^+^ T cells in the TDLN, frozen sections of inguinal LNs 5 days after Ab injection were stained with anti-PD-1 mAb or ER-TR7 to visualize the LN structure. PD-1^+^ cells were primarily located in the interfollicular region (IFR) of rat IgG-treated TDLNs; however, they were primarily found in the medullar region of anti-4-1BB-treated TDLNs (Fig. 3h; left). Moreover, the LN structure visualized with ER-TR7 staining showed that 4-1BB triggering increased the ER-TR7^+^ area, including the area of the IFR and medullar region (Fig. 3h; right), which was statistically significant (Fig. 3i). These results suggest that 4-1BB triggering in vivo increased the number of activated, IFN-γ-producing PD-1^High^CD8^+^ T cells and allowed them to accumulate in the medullar region, thus altering the lymphatic structure.

### 4-1BB-triggered CD8^+^ T cells temporarily accumulate in TDLNs but successfully egress from TDLNs

**Fig. 4:**
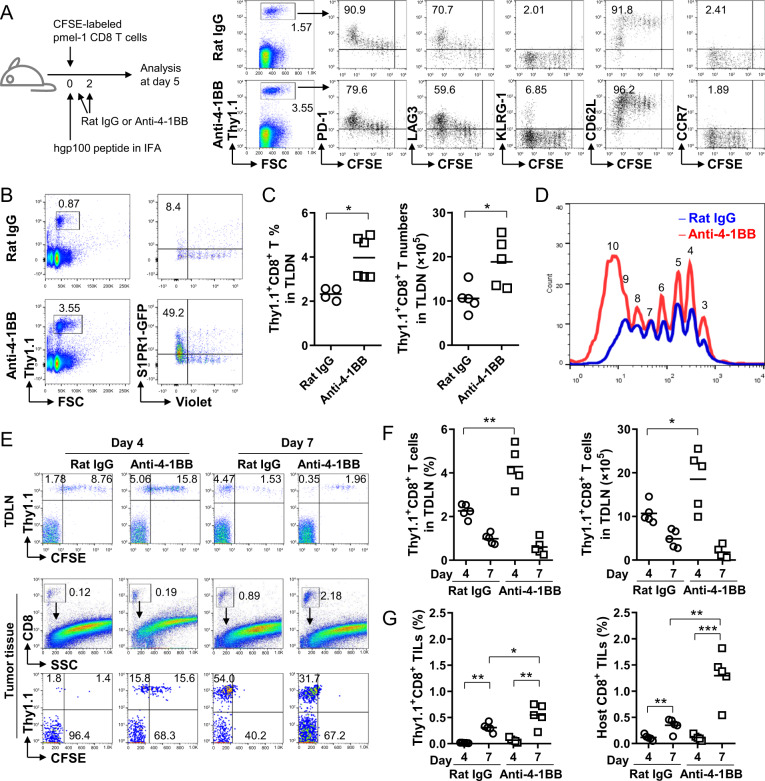
Normal trafficking of pmel-1 CD8^+^ T cells following 4-1BB triggering in vivo. C57BL/6 mice were injected intravenously with CFSE-labeled pmel-1 Thy1.1^+^CD8^+^ T cells (**a**, **c**, **d**) or CellTrace Violet-labeled S1PR1-GFP × pmel-1 Thy1.1^+^CD8^+^ T cells (**b**), immunized with hgp100 peptide in IFA, and further stimulated with rat IgG or anti-4-1BB mAb at days 0 and 2. Inguinal LN cells of the mice that received pmel-1 Thy1.1^+^CD8^+^ T cells were stained with the indicated mAbs at day 5, and gated Thy1.1^+^ cells are plotted CFSE vs. PD-1, LAG3, KLRG-1, CD62L or CCR7 (**a**). Inguinal LN cells of the mice that received S1PR1-GFP × pmel-1 Thy1.1^+^CD8^+^ T cells were stained with anti-Thy1.1-PE on day 5, and gated Thy1.1^+^ cells were plotted CellTrace Violet vs. S1PR1-GFP (**b**). Percentage and absolute number of pmel-1 Thy1.1^+^CD8^+^ T cells in inguinal LNs of the mice that received pmel-1 Thy1.1^+^CD8^+^ T cells at day 5 (**c**). Division rates of pmel-1 Thy1.1^+^CD8^+^ T cells of rat IgG- or anti-4-1BB-treated mice (**d**). **e**–**g** CFSE-labeled naive pmel-1 Thy1.1^+^CD8^+^ T cells were adoptively transferred to B6 mice 7 days after the B16-F10 challenge and simultaneously immunized with hgp100 peptide in IFA. Rat IgG or anti-4-1BB mAb was intraperitoneally injected into the mice on days 7 and 9. The TDLNs and tumor tissues were collected from the mice 4 or 7 days after peptide immunization, counted, and stained with fluorochrome-conjugated anti-Thy1.1, anti-CD8, and anti-CD45 mAbs. Gated CD8^+^ T cells were plotted as Thy1.1 vs. CFSE (**f**). The percentage and absolute number of Thy1.1^+^CD8^+^ T cells in the TDLNs (**g**). The percentage of transferred Thy1.1^+^ and endogenous Thy1.1^−^CD8^+^ T cells in the tumor tissues (**h**). Data are from three (**a**–**d**) and two (**e**–**h**) independent experiments with 5–6 mice per experiment. Student’s *t* test was performed in **b** and shown as the means ± SDs (*p < 0.05; **p < 0.01; ***p < 0.005)

Next, we investigated whether 4-1BB triggering caused abnormalities in the trafficking of activated CD8^+^ T cells. To track the activation, proliferation and egress of Ag-specific CD8^+^ T cells, CFSE-labeled naive pmel-1 Thy1.1^+^CD8^+^ T cells were adoptively transferred into B6 mice, boosted with hgp100 peptide in vivo,^31^ and analyzed to determine their phenotypes related to T cell egress following treatment with rat IgG or anti-4-1BB mAb. Immunization with the hgp100 peptide induced the division of pmel-1 CD8^+^ T cells in inguinal LNs. Expression of PD-1 and LAG3, which are activation markers of T cells, were gradually increased on CD8^+^ T cells and peaked on CFSE^Low^ (>9 divided cells) CD8^+^ T cells (Fig. 4a). KLRG-1 (marker of the latent stage of effector CD8^+^ T cells) expression was only found on some of the CFSE^Low^ CD8^+^ T cells (Fig. 4a). For the egress of effector CD8^+^ T cells, CD62L and CCR7 should be downregulated on CD8^+^ T cells.^30^ We found that the expression level of CD62L was decreased only on some CFSE^Low^ CD8^+^ T cells, and CCR7 rapidly disappeared from CD8^+^ T cells as early as the first division (Fig. 4a). 4-1BB triggering in vivo did not alter the expression pattern of these molecules on CD8^+^ T cells but rather led to the accumulation of all dividing CD8^+^ T cells (Fig. 4a). These data indicate that PD-1^High^CD8^+^ T cells in 4-1BB-triggered inguinal LNs were activated CD8^+^ T cells in proliferation mode, and the downregulation of CCR7 and CD62L, required for the proper egress of activated CD8^+^ T cells, was not compromised by 4-1BB triggering. Moreover, the expression of S1PR1, another key factor for T cell egress, was also normally re-induced on >9 divided CFSE^Low^CD8^+^ T cells, which was further enhanced by 4-1BB triggering (Fig. 4b). Therefore, we conclude that 4-1BB triggering in vivo led to the accumulation of PD-1^High^CD8^+^ T cells in inguinal LNs but did not alter their egress-related phenotype.

4-1BB triggering increased the percentages and absolute numbers of pmel-1 CD8^+^ T cells in inguinal LNs (Fig. 4c) and the accumulation of CFSE^Low^CD8^+^ T cells (Fig. 4d) compared with those in rat IgG-treated mice. Since CD62L downregulation and S1PR1 re-expression, which are essential for egress of activated CD8^+^ T cells, were comparable in both rat IgG- and anti-4-1BB-treated pmel-1 CD8^+^ T cells (Fig. 4a, b), it appears that 4-1BB triggering did not alter the egress function of activated CD8^+^ T cells. Therefore, we next examined whether the activated pmel-1 CD8^+^ T cells ultimately migrated from inguinal TDLNs to tumor tissues following 4-1BB triggering. CFSE-labeled naive pmel-1 Thy1.1^+^CD8^+^ T cells were adoptively transferred into B6 mice 7 days after subcutaneous injection of B16-F10 melanoma cells and boosted with hgp100 peptide in vivo. Immunization with the hgp100 peptide induced the division of pmel-1 CD8^+^ T cells in TDLNs, while 4-1BB triggering accelerated the division of pmel-1 CD8^+^ T cells by increasing the number of CFSE^Low^CD8^+^ T cells on day 4 (Fig. 4e; top). On day 7, pmel-1 CD8^+^ T cells of the CFSE^Low^ phenotype were still abundantly observed in TDLNs of rat IgG-treated mice, while this phenotype was rarely detected in TDLNs of 4-1BB-triggered mice (Fig. 4e; top). Conversely, CFSE^Low^ pmel-1 CD8^+^ T cells were detected in tumor tissues of anti-4-1BB-treated mice from day 4 and further increased at day 7, while they were only found in tumor tissues of rat IgG-treated mice beginning on day 7 (Fig. 4e; bottom). Statistical analysis also demonstrated that the percentage and number of pmel-1 Thy1.1^+^CD8^+^ T cells were significantly increased by anti-4-1BB triggering in TDLNs at day 4 but rapidly decreased over the following 3 days (Fig. 4f). Conversely, tumor infiltration of transferred pmel-1 Thy1.1^+^CD8^+^ T cells gradually increased in rat IgG-treated mice over 7 days, which was further enhanced by 4-1BB triggering (Fig. 4g). Consistent results were observed for the tumor infiltration of endogenous Thy1.1^−^CD8^+^ T cells (Fig. 4g). These results indicate that although 4-1BB triggering caused the accumulation of fully divided pmel-1 CD8^+^ T cells of the CFSE^Low^ phenotype in TDLNs, they eventually egressed from TDLNs and infiltrated into tumor tissues.

Therefore, we conclude that temporal 4-1BB triggering transiently increased the number of activated effector CD8^+^ T cells in TDLNs and induced LN swelling; however, chronic 4-1BB triggering sustained the accumulation of effector CD8^+^ T cells in TDLNs due to the continuous activation of CD8^+^ T cells and thus led to the development of granulomas in TDLNs.

### 4-1BB-induced granulomas in the TDLN impair antitumor CD8^+^ T cell responses

Since granulomas can cause dysfunction of local organs, we next investigated whether 4-1BB-induced granuloma formation could cause defects in the proliferation and trafficking of CD8^+^ T cells in TDLNs. We predicted that granuloma development would have a negative impact on the latter phase of anti-4-1BB therapy; however, the tumor growth of MC38 cells (Fig. 1b) remained significantly suppressed even in the mice chronically treated with anti-4-1BB mAb. This result may have been caused by 4-1BB triggering, inducing granuloma formation after the robust enhancement of antitumor CD8^+^ T cell responses during the early phase of tumor growth. Therefore, it is necessary to monitor the proliferation and tumor infiltration of CD8^+^ T cells in the presence or absence of granuloma formation in TDLNs.

**Fig. 5:**
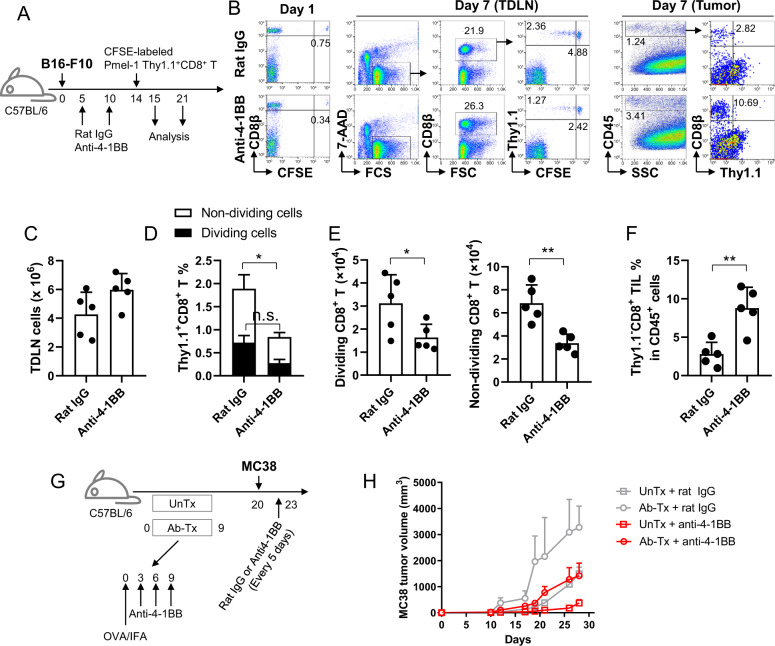
Proliferation and trafficking of pmel-1 CD8^+^ T cells and tumor growth in mice pretreated with anti-4-1BB mAb. **a**–**f** B16-bearing C57BL/6 mice were treated with rat IgG or anti-4-1BB mAb at days 5 and 10 and received CFSE-labeled naive Thy1.1^+^CD8^+^ T cells at day 14. TLDNs and tumor tissues were analyzed at days 15 and 21. **a** Schematic diagram of the experiment. **b** The inguinal TDLNs were collected from the mice 1 or 7 days after CD8^+^ T cell transfer, stained with fluorochrome-conjugated anti-CD8 and anti-Thy1.1 mAb, and further stained with 7-AAD. Single-cell suspensions of tumor tissues were stained with fluorochrome-conjugated anti-CD45, anti-CD8, and anti-Thy1.1 mAbs. All samples were subsequently analyzed by FACSCalibur (BD Bioscience). **c** Total cell numbers at day 21. **d** Percentages of dividing and nondividing pmel-1 Thy1.1^+^CD8^+^ T cells in TDLNs at day 21. **e** Absolute numbers of dividing and nondividing pmel-1 Thy1.1^+^CD8^+^ T cells in TDLNs at day 21. **f** Percentages of Thy1.1^−^CD8^+^ TILs in CD45^+^ cells from tumor tissues at day 21. **g**–**h** C57BL/6 mice were immunized with 20 μg OVA in IFA and injected with anti-4-1BB mAb at days 3, 6, and 9. On day 20, the untreated (UnTx) or the OVA + mAb-treated (Ab-Tx) mice were injected subcutaneously with MC38 tumor cells and further received rat IgG or anti-4-1BB mAb every 5 days from day 3 after the tumor challenge. Tumor growth rates were monitored every 3–4 days. Data are from two (**b**–**f**) or three (**h**) independent experiments with five mice per experiment. Student’s *t* test was performed in **c**–**f** and shown as the means ± SDs (*p < 0.05; **p < 0.01)

To this end, we first induced LN swelling in B16-F10-bearing mice by injecting anti-4-1BB mAb and then transferred CFSE-labeled naive pmel-1 Thy1.1^+^CD8^+^ T cells and monitored the percentages and division rates of pmel-1 CD8^+^ T cells in TDLNs (Fig. 5a). One day after the cell transfer, the percentages of pmel-1 CD8^+^ T cells were lower in anti-4-1BB-treated mice in TDLNs than in rat IgG-treated mice, indicating that homing of naive CD8^+^ T cells was compromised in anti-4-1BB-treated TDLNs. Moreover, on day 7, the division rates of pmel-1 CD8^+^ T cells were lower in anti-4-1BB-treated TDLNs than in rat IgG-treated TDLNs (Fig. 5b). Although the transferred pmel-1 CD8^+^ T cells proliferated in the TDLNs of B16-F10-bearing mice, they were not found in either rat IgG- or anti-4-1BB-pretreated mice, which was likely due to their poor proliferation rate, while endogenous Thy1.1^−^CD8^+^ TILs were more abundantly found in tumor tissues of anti-4-1BB-treated mice compared to those of rat IgG-treated mice (Fig. 5b). Statistical analysis indicated that 4-1BB triggering tended to increase total lymphocyte numbers and decrease the percentage of dividing pmel-1 Thy1.1^+^CD8^+^ T cells in TDLNs; however, these results were not significant (Fig. 5c, d). Moreover, pretreatment with anti-4-1BB mAb significantly reduced the number of both dividing and nondividing pmel-1 Thy1.1^+^CD8^+^ T cells (Fig. 5e) and increased the number of endogenous CD8^+^ TILs (Fig. 5f). Furthermore, IFN-γ production by transferred pmel-1 CD8^+^ T cells was decreased in anti-4-1BB-pretreated TDLNs due to the impaired division rate of pmel-1 CD8^+^ T cells, while endogenous CD8^+^ T cells comparably produced IFN-γ in both rat IgG- and anti-4-1BB-pretreated TDLNs (Supplementary Fig. 5a). Meanwhile, IFN-γ production by endogenous CD8^+^ TILs was enhanced in anti-4-1BB-pretreated mice, with comparable expression of KLRG-1, LAG3, PD-1, and TIGIT on endogenous CD8^+^ TILs from both rat IgG- and anti-4-1BB-pretreated mice (Supplementary Fig. 5a, b). These results indicate that the impaired CD8^+^ T cell response in anti-4-1BB-pretreated mice was restricted to TDLNs rather than tumor tissues.

We next examined whether 4-1BB-mediated antitumor responses were weakened in mice that developed granuloma-like lesions in TDLNs. To this end, C57BL/6 mice were immunized with OVA to induce T cell responses that do not target the tumor cells and treated with anti-4-1BB mAb to develop granuloma-like lesions in the TDLN (Fig. 5g). To minimize the effects of the preinjected Ab, the OVA + mAb-treated (Ab-Tx), and untreated (UnTx) B6 mice were challenged subcutaneously with MC38 tumor cells 11 days after the final injection of Abs and further received rat IgG or anti-4-1BB mAb. In rat IgG-treated mice, the growth rates of MC38 tumors were accelerated in Ab-Tx mice compared with UnTx mice (Fig. 5h), and anti-4-1BB-mediated suppression of tumor growth was attenuated in Ab-Tx mice compared with UnTx mice (Fig. 5h).

Taken together, our data indicate that although 4-1BB triggering enhanced CD8^+^ T cell-mediated antitumor responses in the early phase of treatment, 4-1BB triggering in the latter phase of treatment weakened the antitumor responses by impairing sequential activation of CD8^+^ T cells.

### PD-1 blockade synergistically enhances anti-4-1BB-induced granuloma formation in TDLNs

**Fig. 6:**
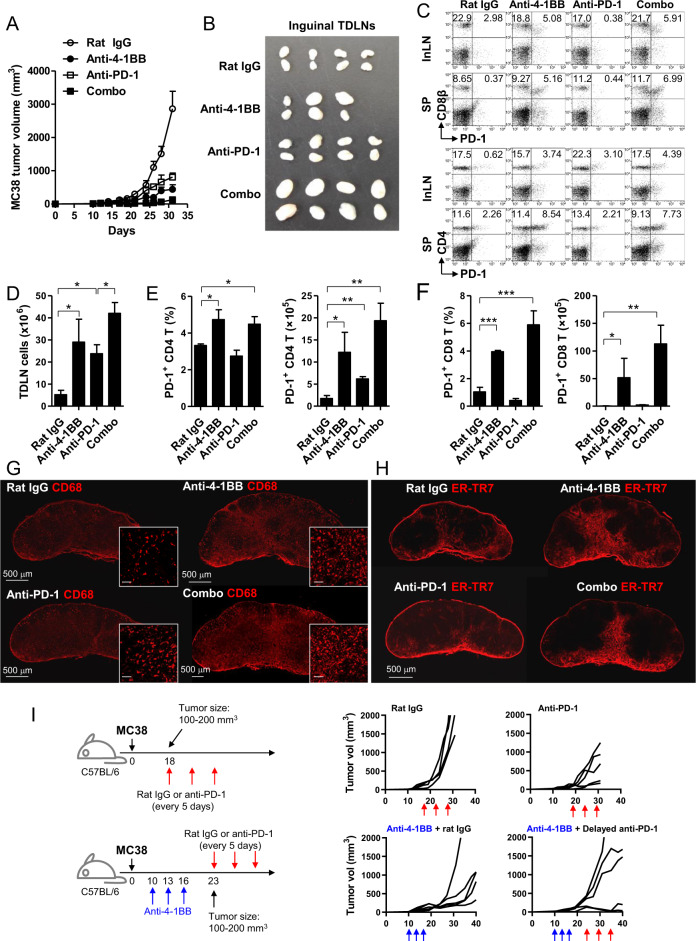
Combined 4-1BB agonist and PD-1 antagonist treatment of MC38 tumor-bearing mice. MC38 tumor-bearing C57BL/6 mice were treated with anti-4-1BB mAb and/or anti-PD-1 mAb every 5 days from day 7 (*n* = 5). **a** Growth rate of MC38 tumors (*n* = 5). **b** The inguinal TDLNs were collected from each group of mice and photographed at day 14 (*n* = 3–4). **c** PD-1 expression on CD4^+^ T and CD8^+^ T cells in the inguinal TDLNs and spleens at day 14. **d** The absolute number of inguinal TDLN cells on day 14. Percentage and absolute number of PD^+^CD4^+^ T cells (**e**), or PD^+^CD8^+^ T cells (**f**) in the inguinal TDLNs. Frozen sections of the inguinal TDLNs at day 14 were stained with anti-mouse CD68-biotin and detected using streptavidin-Cy3 (**g**), or anti-ER-TR7 (BMA) and anti-rat IgG-Cy3 (**h**). Slides were mounted with DAPI-containing solution. Images were captured by a laser scanning microscope (Zeiss LSM780, Carl Zeiss). **i** MC38 tumor-bearing C57BL/6 mice were treated with rat IgG or anti-PD-1 mAb every 5 days from day 18 or first treated with 50 μg of anti-4-1BB mAb every 3 days, for a total of three times, from day 10 and further injected with rat IgG or anti-PD-1 mAb every 5 days, three times, from day 23. All mice were routinely monitored for tumor growth. Data are from two (**g**–**i**) or three (**a**–**f**) independent experiments with five mice (**a**, **g**, **h**, **i**) or 3–4 mice (**b**–**f**) per experiment. Student’s *t* test was performed in **d**–**f** and represented as the mean ± SD (*p < 0.05; **p < 0.01; ***p < 0.005)

Numerous types of mAb-based immunotherapeutic agents designed to enhance antitumor T cell responses are currently in clinical trials and are expected to be used in combination therapies for the treatment of various cancers to maximize their therapeutic effects.^32^ Since repeated 4-1BB triggering led to granuloma development in TDLNs and impaired antitumor T cell responses in the latter phase of anti-4-1BB therapy, we next sought to determine whether 4-1BB-induced development of granulomas in TDLNs is accelerated in the presence of a PD-1 blocker. Therefore, MC38 tumor-bearing mice were treated with a 4-1BB agonist and/or PD-1 antagonist, which resulted in enhanced antitumor immunity (Fig. 6a).^33^ PD-1 blockade alone marginally induced TDLN swelling; however, it served to synergistically accelerate and maximize 4-1BB-induced TDLN swelling (Fig. 6b). Additionally, the number of CD4^+^ and CD8^+^ T cells expressing PD-1 were selectively increased by 4-1BB triggering in the inguinal TDLN and spleen; however, they were not affected by anti-PD-1 mAb treatment alone, while synergistic effects were observed following combined anti-4-1BB mAb and anti-PD-1 mAb treatment (Fig. 6c). The number of TDLN cells also increased by 4–5-fold in response to 4-1BB triggering or PD-1 blockade and by >8-fold following combinatorial treatment (Fig. 6d). Consequently, the proportion and absolute number of PD-1^+^CD4^+^ T cells and PD-1^+^CD8^+^ T cells significantly increased following anti-4-1BB mAb treatment and increased further in combination with anti-PD-1 mAb, while anti-PD-1 mAb alone only significantly increased the number of PD-1^+^CD4^+^ T cells (Fig. 6e, f).

Consistent with these results, CD68 staining of TDLN sections showed that CD68^+^ macrophages were increased by 4-1BB triggering and were further increased following combined PD-1 blockade compared with that in rat IgG-treated mice (Fig. 6g). However, PD-1 blockade alone also had a tendency to increase the number of macrophages (Fig. 6g). Interestingly, the increase in macrophages was primarily located in the IFR, cortical ridges, medullar region, and sinus region rather than in the T or B cell zones (Fig. 6g). Again, ER-TR7^+^ cells were increased by 4-1BB triggering alone or by the 4-1BB agonist plus PD-1 blockade (Fig. 6h).

To investigate whether anti-4-1BB-induced granuloma formation impaired antitumor T cell responses in the latter phase of immunotherapy, MC38 tumor-bearing mice were first treated with anti-4-1BB mAb every 3 days for a total of three times beginning on day 10 to induce LN swelling. To minimize residual anti-4-1BB mAb in the mice, those with 100–200 mm^3^ tumor tissues were selected on day 23 (7 days after last Ab injection) and further treated with rat IgG or anti-PD-1 mAb every 5 days. As a control, MC38 tumor-bearing mice were treated with rat IgG or anti-PD-1 mAb every 5 days from day 18 when the tumor volumes were 100–200 mm^3^. The results show that anti-PD-1 treatment alone moderately suppressed the growth of MC38 tumors compared with that of rat IgG-treated mice (Fig. 6i; upper panel). However, in anti-4-1BB-pretreated mice, MC38 tumor growth was temporally delayed in rat IgG-treated mice, likely due to the lasting effects of anti-4-1BB mAb, and combined treatment with anti-PD-1 led to mixed responses by promoting or synergistically suppressing tumor growth (Fig. 6i; lower panel).

These data indicate that 4-1BB-induced granuloma formation was enhanced in combination with the PD-1 blocker, and thus, immune checkpoint inhibitors that enhance the proliferation of CD8^+^ T cells in TDLNs may have the potential to cause granuloma development in TDLNs. Moreover, since delayed anti-PD-1 treatment was ineffective in suppressing tumor growth in more than half of the anti-4-1BB-pretreated mice (Fig. 6i), we conclude that the enhancement of antitumor CD8^+^ T cell proliferation and the development of granuloma in TDLNs as a feedback inhibitor of the excessively enhanced CD8^+^ T cell responses are two sides of the same coin in controlling overall antitumor immunity.

## Discussion

In the present study, we found that 4-1BB triggering enhanced the proliferation and survival of activated CD8^+^ T cells in vivo and thus led to the accumulation of PD-1^High^CD8^+^ T cells in the medulla of secondary lymphoid organs. PD-1^High^CD8^+^ T cells activated through 4-1BB triggering eventually egressed from TDLNs, infiltrated into tumor tissues, and suppressed tumor growth during the early phase of anti-4-1BB therapy. Since the PD-1^High^CD8^+^ T cells were IFN-γ-producing effector T cells, the repeated injection of anti-4-1BB mAb induced granuloma formation in TDLNs, likely due to the excessive and sustained accumulation of IFN-γ-producing PD-1^+^CD8^+^ T cells and thus impaired sequential activation of CD8^+^ T cells in TDLNs. In general, the excessive triggering of costimulatory molecules, including CD28, CD27, and 4-1BB, resulted in detrimental effects, particularly on B cells, via IFN-γ-producing T cells.^27,34,35^ Our data further demonstrate that chronic activation of 4-1BB has detrimental effects on the immune system by inducing the development of granulomas in TDLNs.

Granulomas are an organized collection of activated immune cells, including macrophages, formed to segregate and destroy invading substances; however, they frequently become pathological in certain infections, including tuberculosis and schistosomiasis.^29^ Moreover, it has been shown that Th1-type immune responses, including those mediated by IFN-γ, lead to activation of myeloid cells and local inflammation and can also cause dysfunction of the local organs.^29,36^ Specifically, sarcoidosis is an inflammatory disease characterized by granuloma in the lung, skin, and LN^36^ and has recently been reported as one of the irAEs in patients successfully treated with PD-1 or CTLA-4 blockers.^8–10,37^ Several studies have reported that sarcoidosis or sarcoid-like reactions in patients who experienced good oncologic responses to treatment disappeared after discontinuation of the drug^8–10,37^ and did not involve caseating necrosis or microorganisms.^10^ This could be due to the uncontrolled activation of T cells, temporally causing the development of granulomas in LNs; however, its underlying mechanisms and implications in cancer patients have not been fully explored. Here, we revealed that granuloma formation in TDLNs is an uncharacterized adverse effect of repeated exposure to agonistic anti-4-1BB mAb and demonstrated that antitumor CD8^+^ T cell responses in vivo could be weakened in the latter phase of anti-4-1BB therapy and by combination with PD-1 blockers (Fig. 6i). Therefore, our findings suggest that immuno-oncology drugs boosting T cell activation in LN, including PD-1 blockade,^4^ can cause granuloma formation in TDLNs if T cells are continuously activated and proliferate in TDLNs. The cancer-immunity cycle explains a series of stepwise events that must be initiated and progress to eradicate cancers.^38^ Since the final goal of cancer immunotherapy is the self-sustenance of the cancer-immunity cycle,^38^ successful treatment of cancers essentially requires the unceasing activation of T cells. If we take the dual aspects of CD8^+^ T cell responses into account, the balance between the unceasing activation of T cells and the formation of granulomas in TDLNs will be crucial in inducing the durable regression of cancers.

Herein, 4-1BB triggering in vivo typically induced excessive swelling of secondary lymphoid organs, including TDLNs, even when it occurred in combination with other immune therapeutics (Fig. 6b), and the accumulation of PD-1^+^CD8^+^ T cells in TDLNs and spleen (Fig. 3a). It seemed that the egress of CD8^+^ T cells was not compromised by 4-1BB triggering, as S1PR1, CD62L, and CCR7 expression was normal in 4-1BB-triggered CD8^+^ T cells (Fig. 4a, b). However, 4-1BB triggering led to the accumulation of dividing CD8^+^ T cells, likely by preventing them from undergoing AICD, and thus caused the massive accumulation of CFSE^Low^CD8^+^ T cells, which divided >9–10 times (Fig. 4c, d). Although CFSE^Low^CD8^+^ T cells with the CD62L^Low^S1PR1^+^ phenotype seemed to be ready to egress from TDLNs (Fig. 4a, b), CFSE^Low^CD8^+^ T cells with the CD62L^High^ phenotype, which were not ready for egress, were still abundant in LNs (Figs. 3c and 4a). We found that when IFN-γ-producing PD-1^+^CD8^+^ T cells temporally accumulated in TDLNs (Fig. 3g), structural alteration of the TDLN was reversed, and granulomas in TDLNs did not develop (Fig. 3f, g). Therefore, it was reasonable to explain the LN swelling and granuloma formation as follows: (1) 4-1BB triggering expands the activated PD-1^High^CD8^+^ T cells by rescuing them from AICD (Figs. 3a, 4a, d); (2) CD8^+^ T cells that divide >9–10 times egress from TDLNs by downregulating CD62L and CCR7 and re-expressing S1PR1 (Fig. 4a, b); (3) repeated injection of anti-4-1BB mAb sustains the accumulation of IFN-γ-producing CD8^+^ T cells with a CD62L^High^ phenotype in medullar of TDLNs and begins to recruit macrophages likely to avoid activated CD8^+^ T cell-mediated tissue damage (Fig. 1h and 3h); and (4) granulomas are developed in the multiple foci of TDLNs (Fig. 1g, h) and impair sequential T cell activation in TDLNs (Fig. 5b, c).

Although we suggest that all ICB-boosting T cell responses in TDLNs, including production of agonistic anti-4-1BB and antagonistic anti-PD-1 mAbs, may have the potential to lead to the development of granulomas in TDLNs due to the excessive accumulation of IFN-γ-producing T cells, recent clinical reports indicate that sarcoidosis in LN adjacent tumor tissues developed in some cancer patients with good oncologic responses to PD-1 blockade,^10,39^ and the repeated injection of anti-4-1BB mAb was generally beneficial to suppressing tumor growth (Fig. 1b), even though the granuloma was developed in inguinal TDLNs (Fig. 1g, h). Given that numerous small and large LNs were involved in inducing antitumor immunity, this dilemma may imply that 4-1BB triggering enhances antitumor CD8^+^ T cell responses in multiple LNs, including major TDLNs such as inguinal LNs and that every LN may differ in the level of granuloma development and impairment of CD8^+^ T cell proliferation. Therefore, overall antitumor immunity could be sufficient to control tumor growth. Indeed, active PET uptake and sarcoidosis in LNs can serve as potential prognostic biomarkers to identify which cancer patients will exhibit good oncologic responses to PD-1 antagonists or 4-1BB agonists during treatment.^40^ However, granuloma development in TDLNs may have contradictory implications due to excessive CD8^+^ T cell responses and subsequent impairment of sequential CD8^+^ T cell responses. Hence, the clinical significance of this observation requires further investigation in clinical settings.

It remains unclear whether granulomas in TDLNs will be found in cancer patients treated with agonistic anti-human 4-1BB mAb. Moreover, since we carried out all experiments with rat IgG reagents rather than mouse IgG reagents, the data from the animal model may be skewed due to the anti-rat responses following the multiple injection of rat IgG Ab and FcR affinity incongruity.^41^ Hence, this discrepancy must be taken into account when translating these data into clinical settings when using human or humanized Abs. Nevertheless, trials are underway to examine the effects of combined coinhibitory receptor blockade and costimulatory receptor agonists, such as 4-1BB and OX40.^42,43^ These combinatorial therapies may serve to increase the chance of developing granulomas in TDLNs, as they are intended to synergistically boost the activation of T cells. In fact, similar to our data, combined treatment with anti-PD-1 and anti-OX40 mAb caused overactive T cell responses, which were deleterious to overall antitumor immune responses.^43^ Therefore, combination immunotherapy may require optimal dosing and timing to maximize benefit and minimize possible toxicity. Moreover, since the balance between the unceasing activation of T cells and granuloma in TDLNs may be crucial for inducing the durable regression of cancers, the occurrence, implication, and long-term impacts of granulomas in the TDLNs of cancer patients receiving ICB, including 4-1BB agonists, need to be further investigated.

## Supplementary information

Supplemental Figures
