# Supplementary material for: Chronic activation of 4-1BB signaling induces granuloma development in tumor-draining lymph nodes that is detrimental to subsequent CD8+ T cell responses
Source: Cell Mol Immunol. 2020 Aug 31;18(8):1956–68. doi: 10.1038/s41423-020-00533-3 (PMC8322392; doi:10.1038/s41423-020-00533-3)
Supplement: Supplementary file 1 — Supplemental Figures [file 41423_2020_533_MOESM1_ESM.docx]

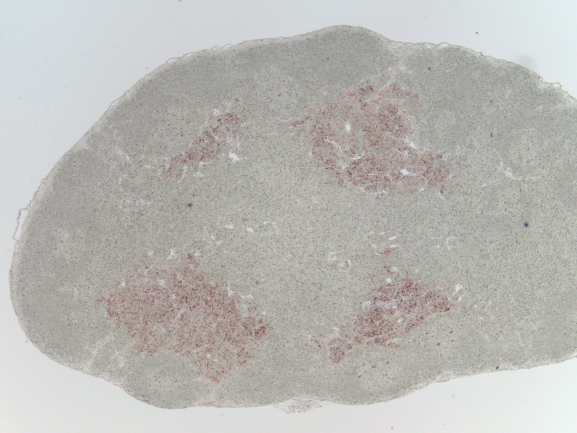

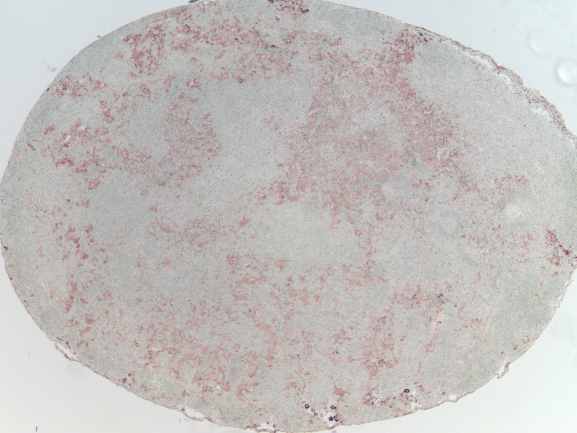

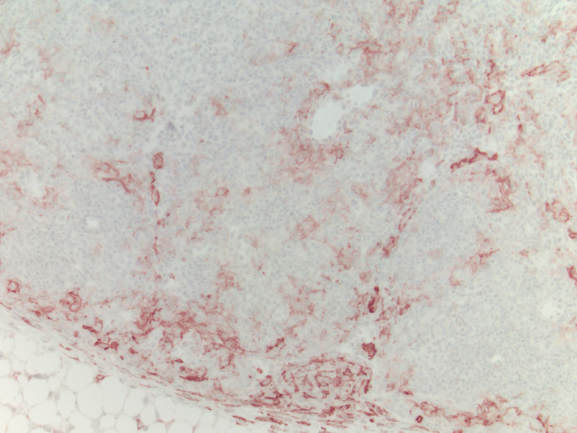

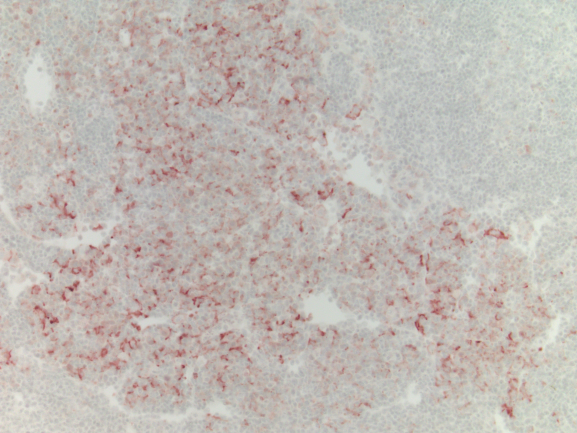


**Rat IgG**

**Anti-4-1BB**

**Supplemental Figure 1. F4/80-positive macrophages in TDLNs of rat IgG- or anti-4-1BB treated mice**

C57BL/6 mice were injected subcutaneously with MC38 tumor cells and received rat IgG, or anti-4-1BB mAb every five days from day 10 for a total of four times. Three days after the 4^th^ mAb injection, TDLNs were collected and fixed with 10% formalin solution. TDLNs were embedded in paraffin wax and 5 mm sections were cut. The slides were stained with anti-F4/80 mAb, and subsequently stained with HRP-conjugated secondary Ab and colorized with DAB chromogen. Data are from two independent experiments with three mice per experiment.


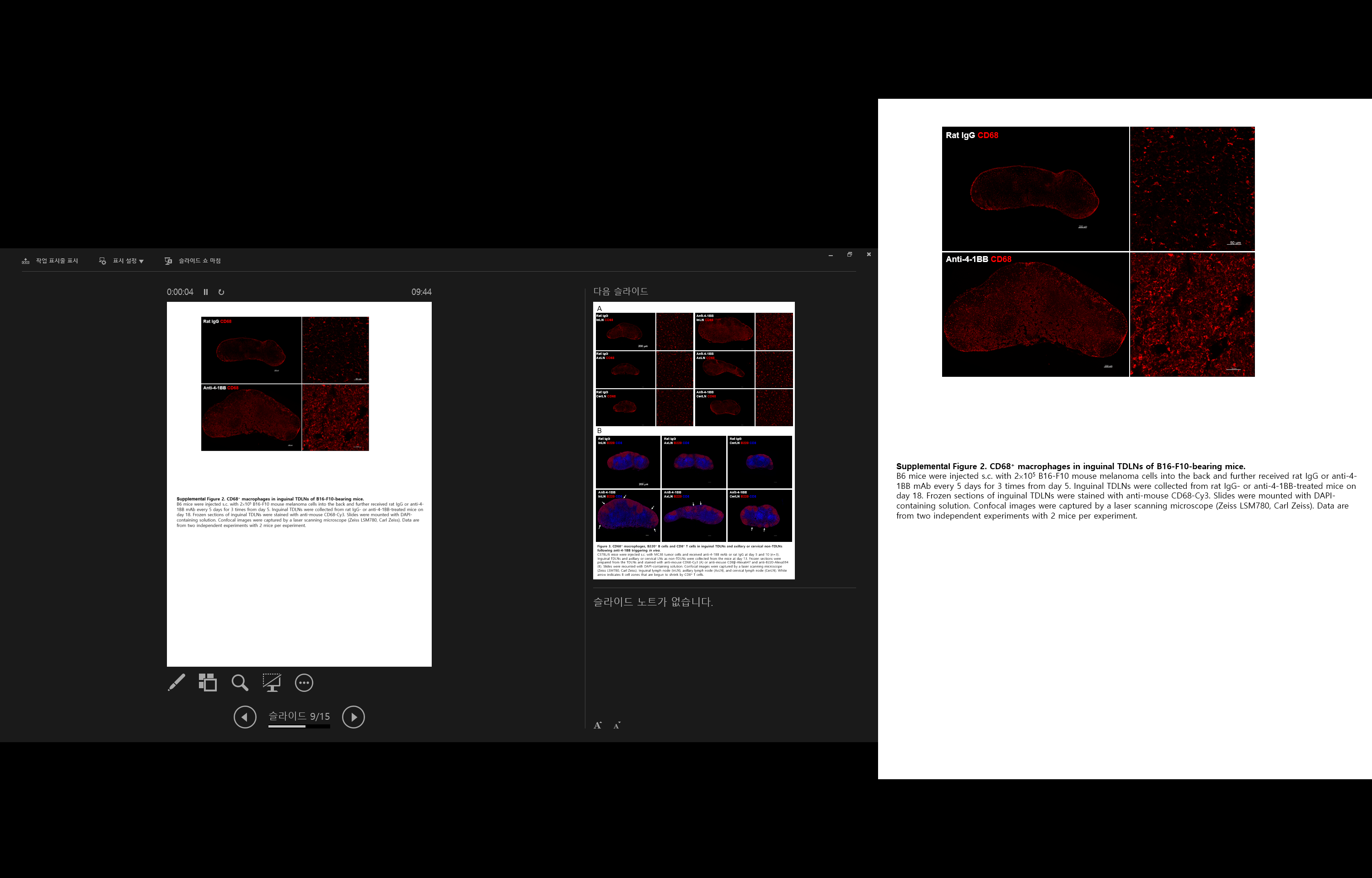


**Supplemental Figure 2. CD68^+^ macrophages in inguinal TDLNs of B16-F10-bearing mice**

B6 mice were injected subcutaneously with 2×10^5^ B16-F10 mouse melanoma cells into the back and further received rat IgG or anti-4-1BB mAb every five days, a total of three times, from day 5. Inguinal TDLNs were collected from rat IgG- or anti-4-1BB-treated mice on day 18. Frozen sections of inguinal TDLNs were stained with anti-mouse CD68-Cy3. Slides were mounted with DAPI-containing solution. Confocal images were captured with a laser scanning microscope (Zeiss LSM780, Carl Zeiss). Data are from two independent experiments with two mice per experiment.


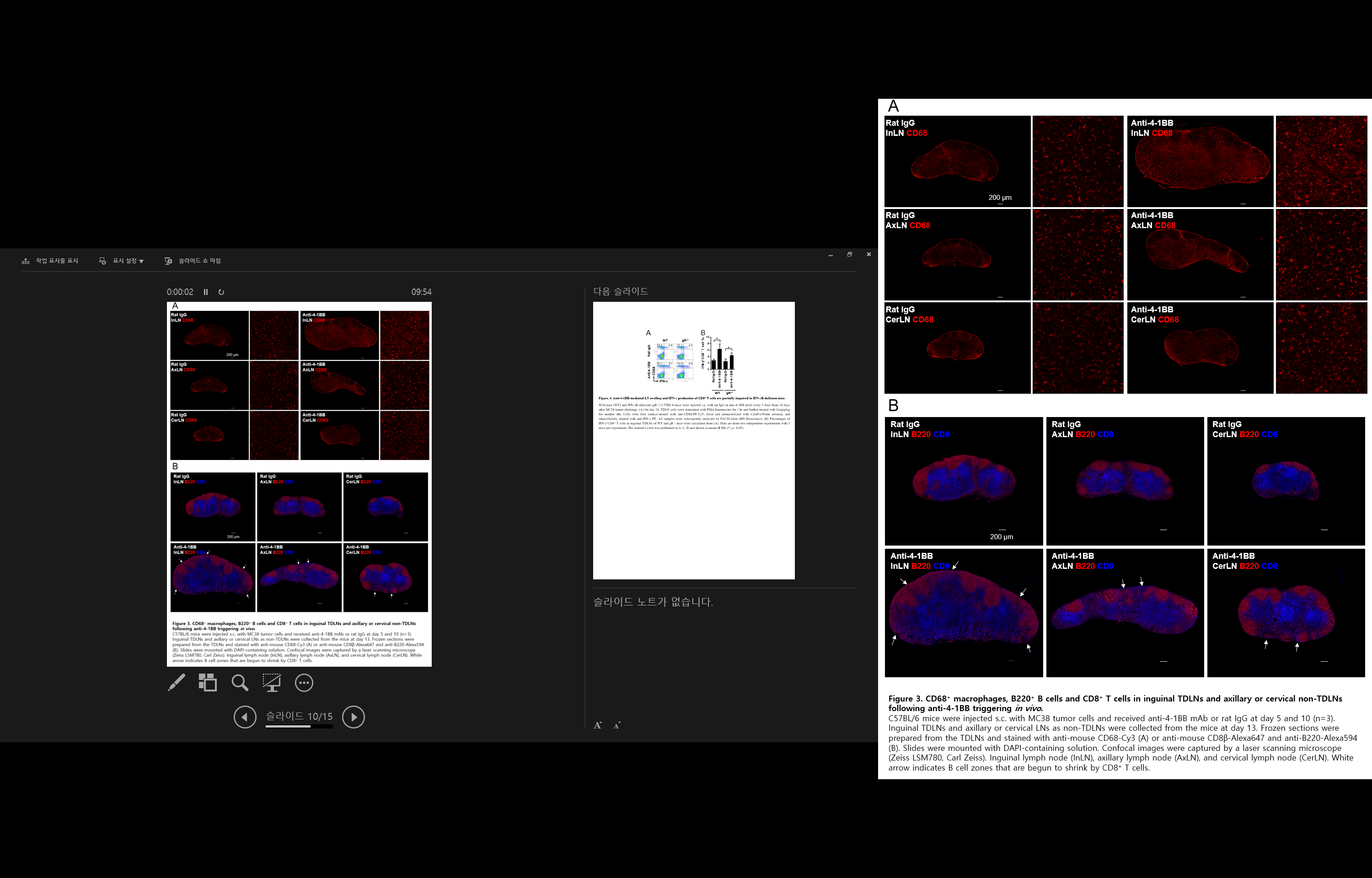


**Supplemental Figure 3.** **CD68^+^ macrophages, B220^+^ B cells and CD8^+^ T cells in inguinal TDLNs and axillary or cervical non-TDLNs following anti-4-1BB triggering *in vivo***

C57BL/6 mice were injected subcutaneously with MC38 tumor cells and received anti-4-1BB mAb or rat IgG at day 5 and 10 (n = 3). Inguinal TDLNs and axillary or cervical LNs as non-TDLNs were collected from the mice at day 13. Frozen sections were prepared from the TDLNs and stained with anti-mouse CD68-Cy3, (A) or anti-mouse CD8β-Alexa647 and anti-B220-Alexa594 (B). Slides were mounted with DAPI-containing solution. Confocal images were captured by a laser scanning microscope (Zeiss LSM780, Carl Zeiss). Inguinal lymph node (InLN), axillary lymph node (AxLN), and cervical lymph node (CerLN). White arrow indicates B cell zones that had begun to shrink by CD8^+^ T cells.


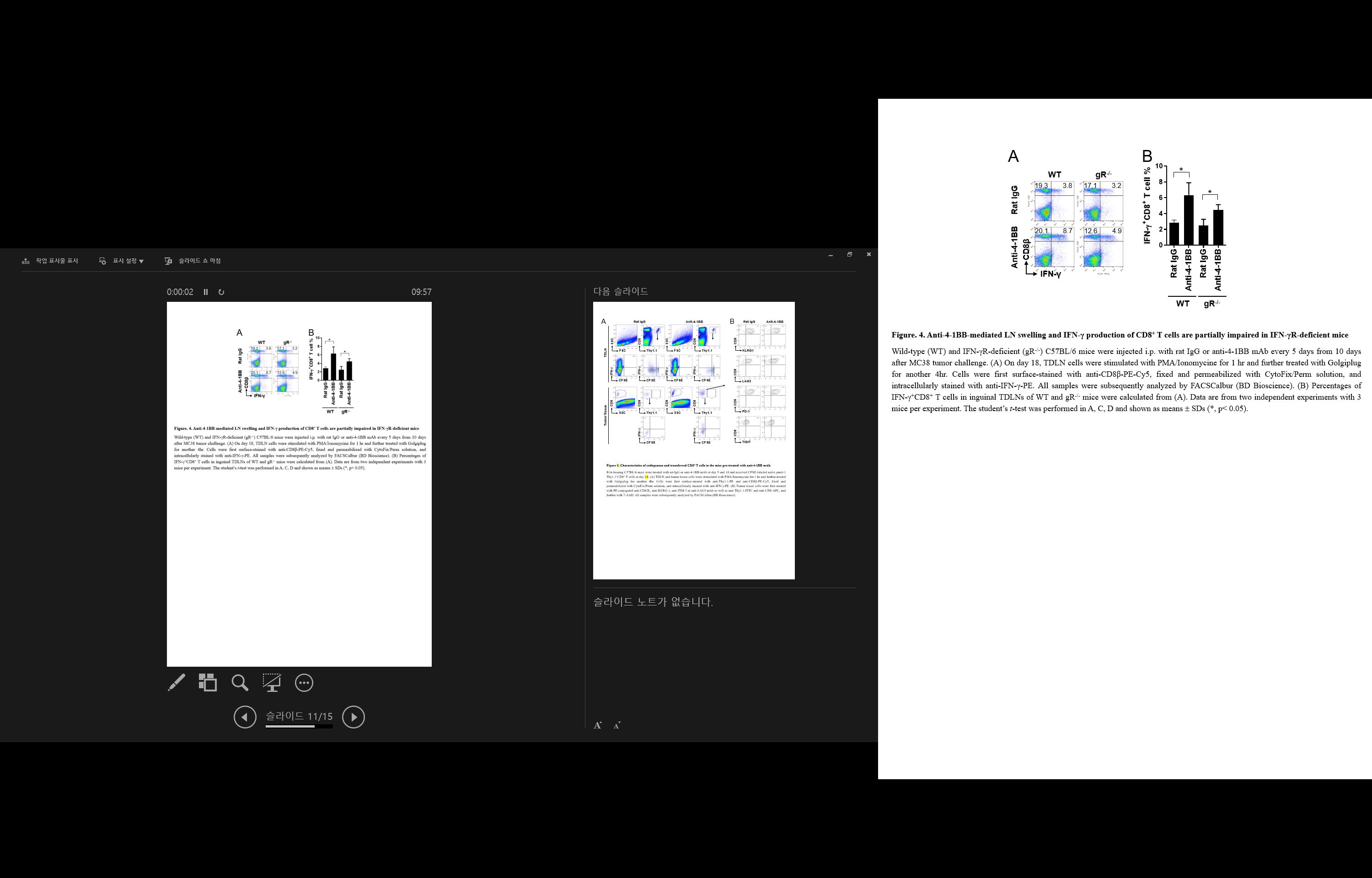


**Supplemental Figure. 4. Anti-4-1BB-mediated LN swelling and IFN-γ production of CD8^+^ T cells are partially impaired in IFN-γR-deficient mice**

Wild-type (WT) and IFN-γR-deficient (gR^-/-^) C57BL/6 mice were injected intraperitoneally with rat IgG, or anti-4-1BB mAb every five days from ten days after MC38 tumor challenge. (A) On day 18, TDLN cells were stimulated with PMA/Ionomycine for 1 h and further treated with Golgiplug for an additional 4 h. Cells were first surface-stained with anti-CD8β-PE-Cy5, fixed and permeabilized with CytoFix/Perm solution, and intracellularly stained with anti-IFN-γ-PE. All samples were subsequently analyzed by FACSCalbur (BD Bioscience). (B) Percentages of IFN-γ^+^CD8^+^ T cells in inguinal TDLNs of WT and gR^-/-^ mice were calculated from (A). Data are from two independent experiments with three mice per experiment. The student’s *t*-test was performed in A, C, D and shown as mean ± SD (* p < 0.05).


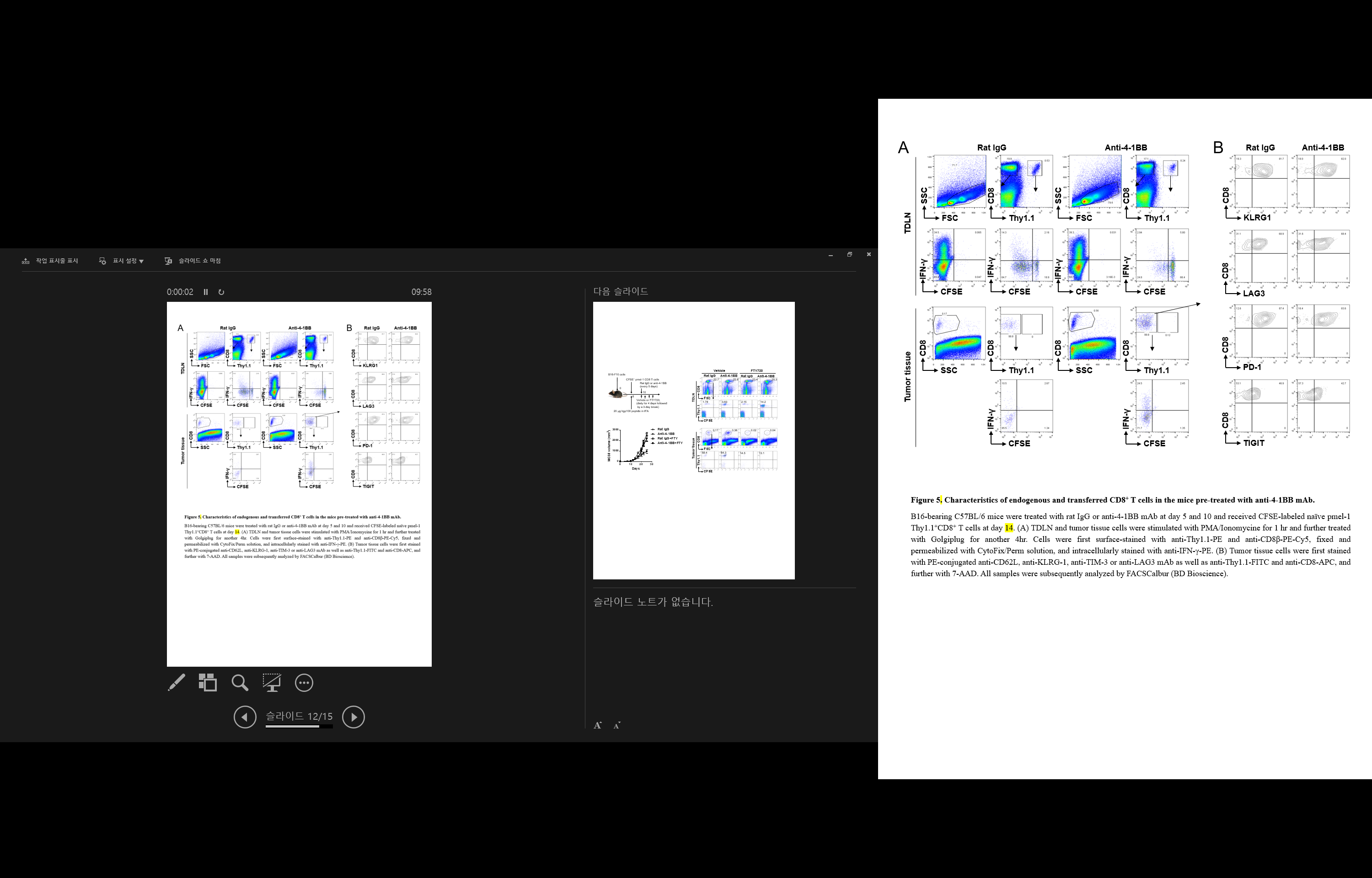


**Supplemental Figure 5. Characteristics of endogenous and transferred CD8^+^ T cells in mice pretreated with anti-4-1BB mAb**

B16-bearing C57BL/6 mice were treated with rat IgG or anti-4-1BB mAb at day 5 and 10 and received CFSE-labeled naïve pmel-1 Thy1.1^+^CD8^+^ T cells at day 14. (A) TDLN and tumor tissue cells were stimulated with PMA/Ionomycine for 1 h and further treated with Golgiplug for an additional 4 h. Cells were first surface-stained with anti-Thy1.1-PE and anti-CD8β-PE-Cy5, fixed and permeabilized with CytoFix/Perm solution, and intracellularly stained with anti-IFN-γ-PE. (B) Tumor tissue cells were first stained with PE-conjugated anti-CD62L, anti-KLRG-1, anti-TIM-3 or anti-LAG3 mAb, as well as with anti-Thy1.1-FITC, and anti-CD8-APC, and further with 7-AAD. All samples were subsequently analyzed by FACSCalbur.
